# Quantified assessment of deep brain stimulation on Parkinson’s patients with task fNIRS measurements and functional connectivity analysis: a pilot study

**DOI:** 10.1186/s41016-021-00251-3

**Published:** 2021-07-05

**Authors:** Ningbo Yu, Siquan Liang, Jiewei Lu, Zhilin Shu, Haitao Li, Yang Yu, Jialing Wu, Jianda Han

**Affiliations:** 1 College of Artificial Intelligence, Nankai University, Tianjin, China; 2 Tianjin Key Laboratory of Intelligent Robotics, Nankai University, Tianjin, China; 3 Department of Neurosurgery, Tianjin Huanhu Hospital, Tianjin, China; 4 Department of Neurorehabilitation, Tianjin Huanhu Hospital, Tianjin, China; 5 Department of Neurology, Tianjin Huanhu Hospital, Tianjin, China; 6 Laboratory of Cerebral Vascular and Neurodegenerative Diseases, Tianjin Neurosurgical Institute, Tianjin Huanhu Hospital, Tianjin, China

**Keywords:** Deep brain stimulation programming, Parkinson’s disease, Brain efficiency, Functional connectivity

## Abstract

**Background:**

Deep brain stimulation (DBS) has proved effective for Parkinson’s disease (PD), but the identification of stimulation parameters relies on doctors’ subjective judgment on patient behavior.

**Methods:**

Five PD patients performed 10-meter walking tasks under different brain stimulation frequencies. During walking tests, a wearable functional near-infrared spectroscopy (fNIRS) system was used to measure the concentration change of oxygenated hemoglobin (△*HbO*_2_) in prefrontal cortex, parietal lobe and occipital lobe. Brain functional connectivity and global efficiency were calculated to quantify the brain activities.

**Results:**

We discovered that both the global and regional brain efficiency of all patients varied with stimulation parameters, and the DBS pattern enabling the highest brain efficiency was optimal for each patient, in accordance with the clinical assessments and DBS treatment decision made by the doctors.

**Conclusions:**

Task fNIRS assessments and brain functional connectivity analysis promise a quantified and objective solution for patient-specific optimization of DBS treatment.

**Trial registration:**

Name: Accurate treatment under the multidisciplinary cooperative diagnosis and treatment model of Parkinson’s disease. Registration number is ChiCTR1900022715. Date of registration is April 23, 2019.

## Background

Parkinson’s disease (PD) is a neurodegenerative disease caused by the progressive loss of nigrostriatal dopaminergic neurons in substantia nigra pars compacta [1]. The loss of dopaminergic neurons induces severe motor symptoms such as tremor, rigidity, bradykinesia and dyskinesia, as well as non-motor symptoms such as constipation, fatigue, anxiety, cognitive dysfunction, and dementia [2, 3]. Deep brain stimulation (DBS) has proved an effective therapy for symptom improvement after PD, especially in the late stage when medication is less effective [4, 5].

**Fig. 1:**
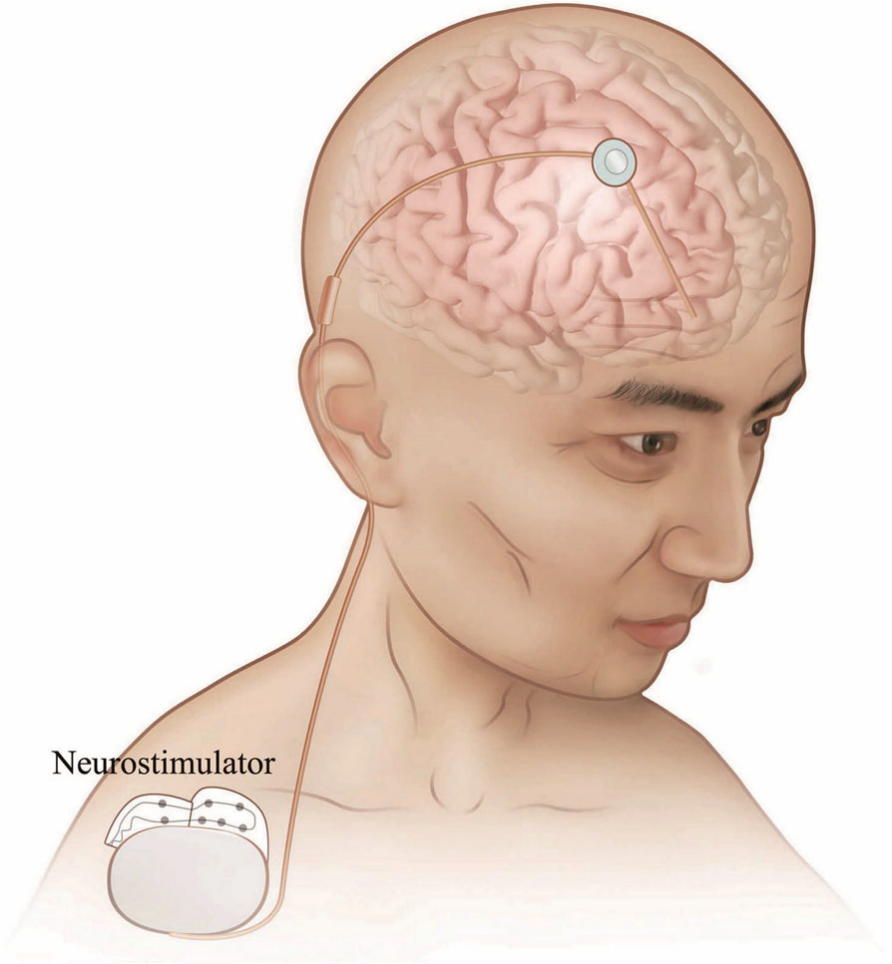
Deep brain stimulation: neurologists implant a neurostimulator to send electrical stimulation through the implanted electrodes to specific targets. After neurosurgery, DBS programming was conducted to optimize the stimulation parameters for the patient

With DBS operation, electrodes are implanted into specific target brain locations, such as the subthalamic nucleus or globus pallidus internal, as shown in Fig. 1. The electrodes work as the neurostimulator and send electrical stimulation for the treatment of movement disorders [6, 7]. DBS programming, the identification of stimulation parameters of the implanted neurostimulator for symptom management, is crucial for successful and optimal treatment. However, the functioning neural mechanism of DBS remains unclear, and DBS programming in current clinical practice is typically conducted by doctors according to their observation of patient behavior, strongly relying on the doctors’ skill, experience, and subjective judgment [8–10]. An objective approach that uses personalized neurophysiological measurements to optimize DBS programming is therefore highly demanded.

As a neurodegenerative disease, PD damages the central nervous system and its function, leading to movement disorders. While DBS improves motor symptoms, changes with respect to brain function definitely happen. Brain functional connectivity (BFC), which refers to the statistical correlation between physiological signals from PD-related brain regions, might characterize DBS-induced functional variation and provide a quantified and objective measure for DBS treatment. Different brain regions communicate and coordinate during function fulfillment and task execution, and brain functional connectivity represents this effort [11–13]. Algorithms have been developed for BFC analysis of neurological disorders [14, 15]. With the DBS treatment, the BFC strength indicates the “cost” of the brain while the patient trying to complete a specific task. The lower the cost, the higher communication efficiency among the brain regions, and the better the DBS parameters might be. In this paper, we verify this idea by clinical tests of PD patients.

To investigate the brain activation of PD patients, previous studies have applied SPECT [16, 17], PET[18], fMRI[19, 20], and EEG [21, 22], mainly by comparison between patients and the healthy controls for diagnosis, but none for DBS programming. Moreover, none of fMRI, SPECT, or PET could allow “task-state” measurements while the patient trying to complete a motor task, and the preparation process for EEG measurement is tedious and challenging for PD patients after DBS surgery, restricting the possibilities for these imaging modalities to be applied in DBS programming. Instead, functional near-infrared spectroscopy (fNIRS) is an optical functional neuroimaging technique that uses near-infrared light (700–900 nm spectral interval) to perform continuous and non-invasive monitoring of blood hemoglobin changes related to brain functions [23]. The fNIRS-based functional connectivity has been successfully used in clinical applications. In [24], Qitao et al. assessed resting-state functional connectivity in cerebral infarction patients with fNIRS. In [25], Didem et al. used functional connectivity features to perform clinical binary classification in patients with fibromyalgia. A further advantage for our study is that the fNIRS equipment can be made portable, enabling “task-state” measurements during clinical evaluation such as walking.

With a wearable fNIRS system, we measured five PD patients in the clinical DBS programming process, recording their brain activities during standard walking tests under different brain stimulation frequencies that were specified by the doctors. The fNIRS-based brain functional connectivity analysis was conducted on three brain regions: the prefrontal cortex, parietal lobe, and occipital lobe. The coordination and communication among these brain regions are essentially evolved during the walking test in clinical DBS programming and their functional connectivity can characterize DBS-induced improvement of neural function.

## Methods

### Participants

The patients of consideration were clinically diagnosed idiopathic Parkinson’s disease without other plus syndromes. Exclusion criteria for patient recruitment include the following: (1) unnecessary to re-adjust the stimulation parameters; (2) unable to stand or walk for 90 s at a time; (3) any factors affecting their gait performance, such as idiopathic scoliosis and leg injury; (4) any mental diseases, such as neuropsychiatric comorbidity, schizophrenia, and personality disorders; and (5) age > 70 years. Five PD patients that received DBS surgery and qualified for the study were recruited. These PD patients had bilateral symptoms and were treated with bilateral stimulation. Each patient was fully informed of the experimental purpose and procedures and provided written consent prior to the measurement. The clinical characteristics and initial stimulation parameters in the DBS surgery of these patients are shown in Table 1.

**Table 1:** Clinical characteristics of the five PD patients for DBS programming

Patient	Age	Gender	Stimulated target	Insertion time ^*a*^	Medication	Hoehn-Yahr	MDS-UPDRS III	MOCA
					duration	Scale		
P1	61	Male	Subthalamic nucleus	12 months	5 Years	3	36	21
P2	58	Female	Subthalamic nucleus	11 months	16 Years	4	102	24
P3	58	Female	Subthalamic nucleus	6 months	5 Years	3	42	24
P4	60	Female	Subthalamic nucleus	8 months	11 Years	3	52	24
P5	63	Female	Subthalamic nucleus	6 months	6 Years	3	60	22

^*a*^ Insertion time: the time after the DBS electrode insertion

### Functional near-infrared spectroscopy

**Fig. 2:**
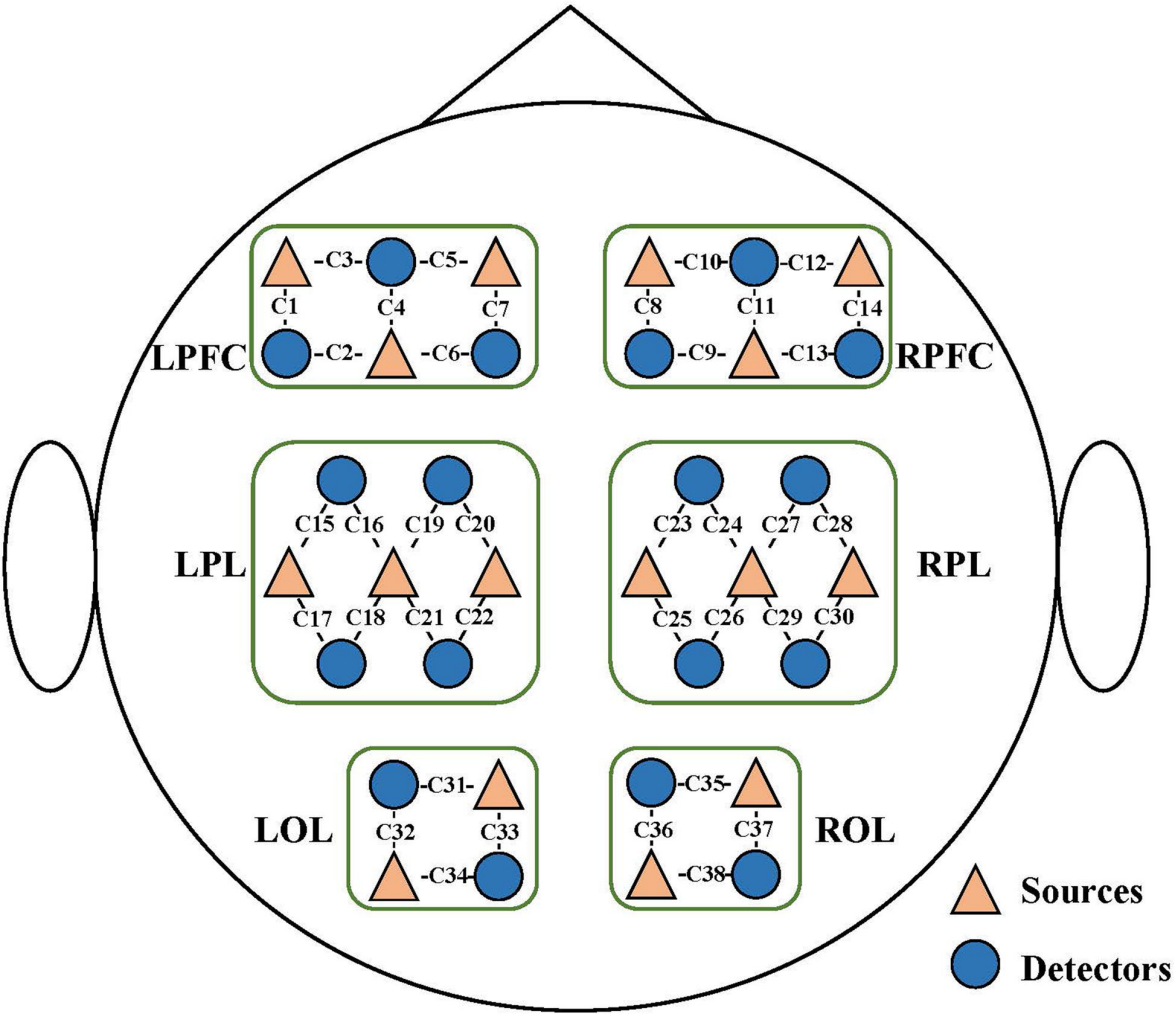
Deployment of the fNIRS sources (16 orange rectangles) and detectors (18 blue dots). C*i* indicates the *i*th channel. The six brain regions of interest, i.e., L/R-PFC, L/R-PL, and L/R-OL, are separated by green frames

A wearable, wireless, continuous-wave fNIRS system (Nirsmart, Danyang Huichuang Medical Equipment Co, Ltd, China) [26] was used to monitor the concentration change of oxygenated hemoglobin (△*HbO*_2_). The wavelength of the near-infrared light was 760 nm, and the sampling rate was 10 Hz. Six regions, i.e., left and right prefrontal cortex (L/R-PFC), parietal lobe (L/R-PL), and occipital lobe (L/R-OL) were chosen as the areas of interest for recording, and 34 fNIRS electrodes including 16 sources and 18 detectors were placed to the selected region, as shown in Fig. 2. The prefrontal cortex is implicated in cognitive control and information processing for complex behavior. The parietal lobe plays an important role in motor function, working memory, and the integration of multiple sensory information. The occipital lobe is responsible for visual processing, working memory, and modulation of different sensory stimulation.

### Experimental design

**Fig. 3:**
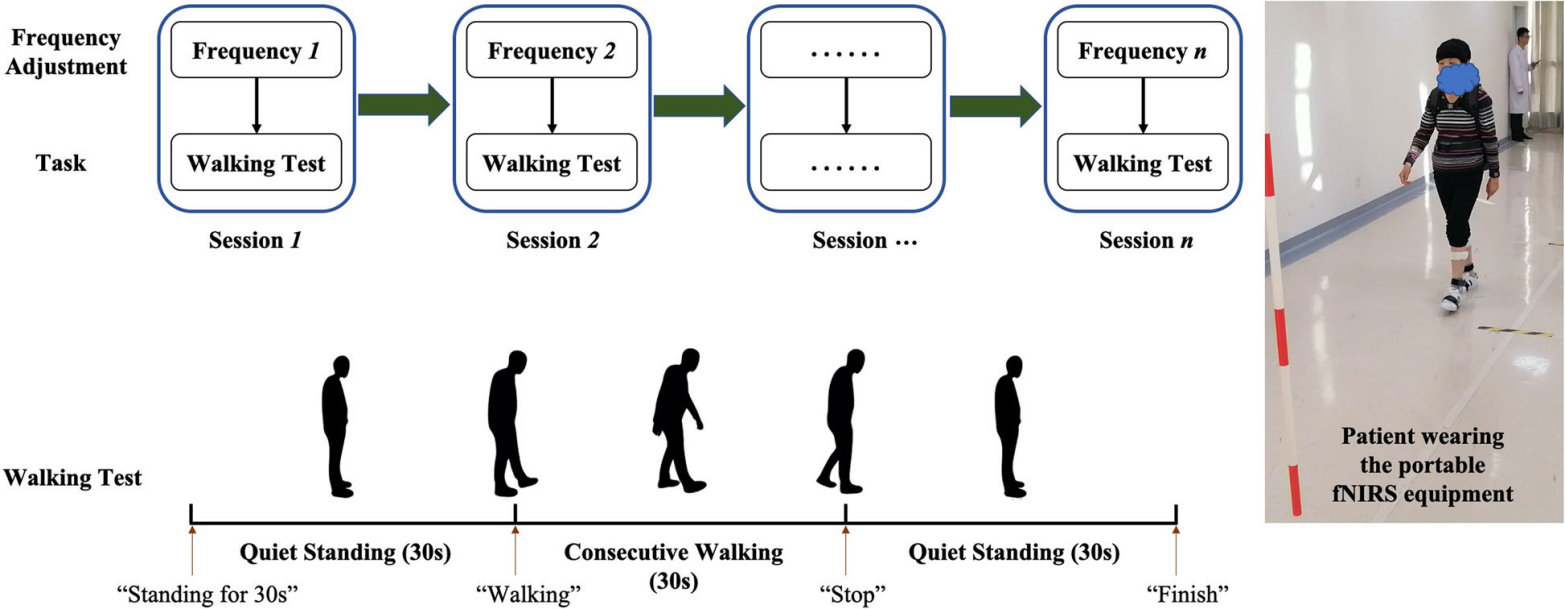
The experimental procedure. Left: each DBS patient performed *n* test sessions, *n* is the number of frequency adjustment. Each session contains frequency adjustment and walking test. The walking test involves 30 s of quiet standing at the beginning and end, intermediated with 30 s of consecutive walking. Instructions about the walking test ("Standing for 30 s," "Walking," "Stop," and "Finish") were given by the doctor. Right: the patients wore a portable fNIRS measurement equipment

The crucial brain stimulation parameters in DBS programming consist of the location of electrode contact, voltage amplitude, and frequency. In our experiments, the DBS frequency was varied while the location of electrode contact and voltage amplitude were invariant. The reasons of only varying DBS frequency included the following: (1) the locations of electrode contact had been optimized by doctors with MRI scans and 3D reconstruction technique. The voltage amplitudes were fixed by the doctors with clinical diagnosis. (2) DBS frequency was related to the improvement of gait and balance [27–30]. After varying the DBS frequency, the patients performed the walking test, as shown in Fig. 3. It has to be noted that DBS patients are normally not strong enough to take too many walking tests. For the five PD patients of this study, we limited the number of walking tests to be 4 at most. The process of the walking test was as follows: (1) the doctor performed the frequency adjustment, (2) the patient sat on a chair for 5 min to ensure that the new DBS paradigm actually took effect, (3) the patient stood up from the chair and stood quite for 30 s, (4) the patient performed the 10-m walking task, and (5) the patient stood quite for 30 s again. Instructions of “Standing,” “Walking,” “Stop,” and “Finish” were given by the doctors during each test. PD patients were tested under medicine off condition.

### Data processing

#### Preprocessing

Firstly, the collected fNIRS measurement data were processed with a 0.01–0.2 bandpass filter to remove the instrumental and physiological noises (e.g., heartbeats, respirations and Mayer waves) [31–33]. Then, the △*HbO*_2_ of each channel was calculated with the filtered data according to the modified Beer-Lambert law [34]. Further, motion artifacts were removed based on moving standard deviation and spline interpolation [35].

#### Functional connectivity and global efficiency

Firstly, the Pearson’s correlation coefficient $\phantom {\dot {i}\!}P_{C_{x}C_{y}}$ between two channels was calculated as follows:

1 $$ \begin{aligned} P_{C_{x}C_{y}}&=\frac{cov(C_{x},C_{y})}{\sigma_{C_{x}}\sigma_{C_{y}}}\\ &=\frac{\Sigma_{i=1}^{m}(C_{x,i}-\bar{C_{x}})(C_{y,i}-\bar{C_{y}})}{\sqrt{\Sigma_{i=1}^{m}(C_{x,i}-\bar{C_{x}})^{2}}\sqrt{\Sigma_{i=1}^{m}(C_{y,i}-\bar{C_{y}})^{2}}} \end{aligned}  $$

where *C*_*x*_ and *C*_*y*_ are the measurements (△*HbO*_2_) of the *x*th and *y*th channels, *cov*(*C*_*x*_,*C*_*y*_) is the covariance between *C*_*x*_ and $\phantom {\dot {i}\!}C_{y}, \sigma _{C_{x}}$ and $\phantom {\dot {i}\!}\sigma _{C_{y}}$ are the standard deviation of *C*_*x*_ and *C*_*y*_, *m* is the length of the measurement, *C*_*x*,*i*_ and *C*_*y*,*i*_ are the *i*th measurement (△*HbO*_2_) of the *x*th and *y*th channels, and $\bar {C_{x}}=\frac {1}{m}\sum _{i=1}^{m}C_{x,i}$ and $\bar {C_{y}}=\frac {1}{m}\sum _{i=1}^{m}C_{y,i}$ are the average values of *C*_*x*_ and *C*_*y*_.

Then, Fisher’s z-transformation was applied to decrease the skewness of $\phantom {\dot {i}\!}P_{C_{x}C_{y}}$ and normalize its distribution:

2 $$ F_{C_{x}C_{y}} = artanh(P_{C_{x}C_{y}})=\frac{1}{2}ln\left(\frac{1+P_{C_{x}C_{y}}}{1-P_{C_{x}C_{y}}}\right)  $$

where $\phantom {\dot {i}\!}F_{C_{x}C_{y}}$ is the connection strength between channel *C*_*x*_ and *C*_*y*_, and *artanh*(·) is the inverse hyperbolic tangent function. The $\phantom {\dot {i}\!}F_{C_{x}C_{y}}$ values were used to construct the connectivity matrix *M*, which is defined as follows:

3 $$ M = \left[\begin{array}{cccc} F_{C_{N}C_{1}} & F_{C_{N}C_{2}}& \cdots & F_{C_{N}C_{N}}\\ \vdots & \vdots & \ddots & \vdots \\ F_{C_{2}C_{1}} & F_{C_{2}C_{2}} & \cdots & F_{C_{2}C_{N}}\\ F_{C_{1}C_{1}} & F_{C_{1}C_{2}} & \cdots & F_{C_{1}C_{N}} \end{array}\right]  $$

where *N* is the channel number of global brain regions. It should be noted that higher connection strength in the connectivity matrix M corresponds to lower brain communication efficiency [36–38].

Further, the global efficiency (*GE*) describes the overall communication efficiency:

4 $$ GE = \frac{1}{\frac{1}{N} \sum_{x=1}^{N} \frac{\sum_{y=1,y\neq x}^{N}F_{C_{x}C_{y}}}{N-1}}  $$

where *N* is the channel number of global brain regions. *C*_*x*_ and *C*_*y*_ indicate the measurement (△*HbO*_2_) of the *x*th and *y*th channels. $\phantom {\dot {i}\!}F_{C_{x}C_{y}}$ indicates the connection strength between channel *C*_*x*_ and *C*_*y*_. Higher *GE* scores represent lower communication strength of global regions.

#### Local strength

Local strength represents the communication strength between different brain regions. The local strength of PFC, PL, and OL are respectively defined as *LS*_*PFC*_,*LS*_*PL*_, and *LS*_*OL*_, i.e.,

5 $$ \begin{aligned} {LS}_{PFC} &= \frac{1}{2}\left(\frac{1}{N_{LPFC}\times N} \sum_{x=1}^{N_{LPFC}} \sum_{y=1,y\neq x}^{N} F_{C_{x}C_{y}} \right.\\ &+\left. \frac{1}{N_{RPFC}\times N} \sum_{x=1}^{N_{RPFC}} \sum_{y=1,y\neq x}^{N} F_{C_{x}C_{y}}\right) \end{aligned}  $$

6 $$ \begin{aligned} {LS}_{PL} &= \frac{1}{2}\left(\frac{1}{N_{LPL}\times N} \sum_{x=1}^{N_{LPL}} \sum_{y=1,y\neq x}^{N} F_{C_{x}C_{y}} \right.\\ &+\left. \frac{1}{N_{RPL}\times N} \sum_{x=1}^{N_{RPL}} \sum_{y=1,y\neq x}^{N}F_{C_{x}C_{y}}\right) \end{aligned}  $$

7 $$ \begin{aligned} {LS}_{OL} &= \frac{1}{2}\left(\frac{1}{N_{LOL}\times N} \sum_{x=1}^{N_{LOL}} \sum_{y=1,y\neq x}^{N} F_{C_{x}C_{y}}\right. \\ &+\left. \frac{1}{N_{ROL}\times N} \sum_{x=1}^{N_{ROL}} \sum_{y=1,y\neq x}^{N}F_{C_{x}C_{y}}\right) \end{aligned}  $$

where *N* is the channel number of global brain regions. *N*_*LPFC*_,*N*_*RPFC*_,*N*_*LPL*_,*N*_*RPL*_,*N*_*LOL*_, and *N*_*ROL*_ indicate the channel number of LPFC, RPFC, LPL, RPL, LOL, and ROL, respectively. Moreover, the averaged local strength (*L*_*aver*_) is defined as follows:

8 $$ L_{aver} = \frac{N_{PFC}}{N}\times L_{PFC}^{\prime} + \frac{N_{PL}}{N}\times L_{PL}^{\prime} + \frac{N_{OL}}{N}\times L_{OL}^{\prime}  $$

where *N*_*PFC*_,*N*_*PL*_ and *N*_*OL*_ indicate the channel number in PFC, PL, and OL. $\phantom {\dot {i}\!}L_{PFC}^{\prime }, L_{PL}^{\prime }$, and $\phantom {\dot {i}\!}L_{OL}^{\prime }$ represent the normalized *L*_*PFC*_,*L*_*PL*_, and *L*_*OL*_, respectively. $\phantom {\dot {i}\!}L_{PFC}^{\prime }, L_{PL}^{\prime }$, and $\phantom {\dot {i}\!}L_{OL}^{\prime }$ are defined as follows:

9 $$ \small L_{PFC}^{'} = \frac{L_{PFC}-L_{PFC}^{min}}{L_{PFC}^{m}ax-L_{PFC}^{min}}  $$

10 $$ \small L_{PL}^{'} = \frac{L_{PL}-L_{PL}^{min}}{L_{PL}^{m}ax-L_{PL}^{min}}  $$

11 $$ \small L_{OL}^{'} = \frac{L_{OL}-L_{OL}^{min}}{L_{OL}^{m}ax-L_{OL}^{min}}  $$

where $L_{PFC}^{min}, L_{PL}^{min}$ and $L_{OL}^{min}$ indicate the minimum values of *L*_*PFC*_,*L*_*PL*_ and *L*_*OL*_ of each PD patient. $L_{PFC}^{max}, L_{PL}^{max}$ and $L_{OL}^{max}$ indicate the maximum values of *L*_*PFC*_,*L*_*PL*_, and *L*_*OL*_ of each PD patient.

## Results

### Brain functional connectivity and global efficiency

The measurements of fNIRS during the 10-m walking of each patient were recorded, and the brain connectivity matrix (*CM*) as well as the global efficiency (*GE*) were calculated with respect to each tested frequency.

**Fig. 4:**
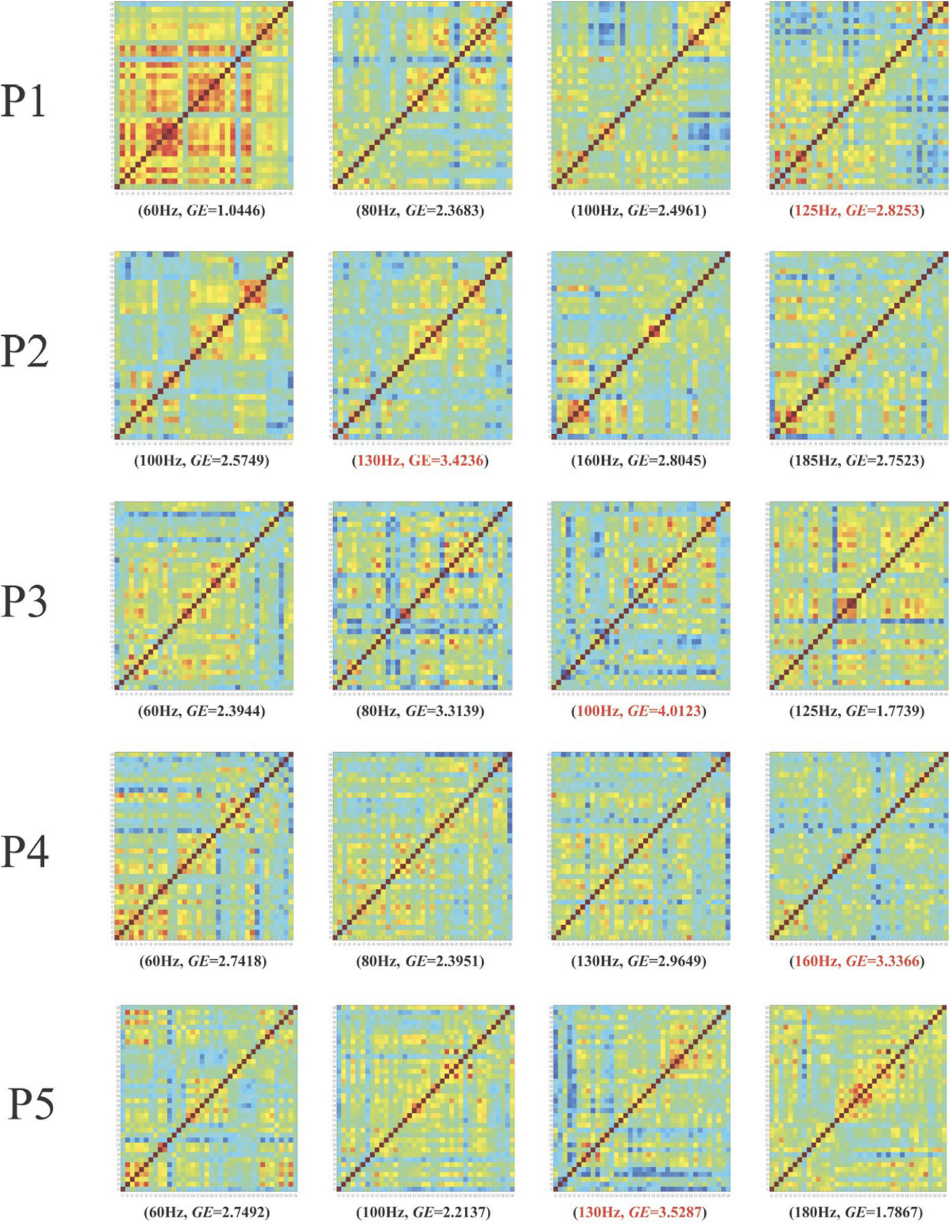
The brain functional connectivity matrices calculated from the fNIRS measurements of the five PD patients (P1, P2, P3, P4, P5) at different DBS frequencies. The color bar corresponding to the values of *CM* was presented at the bottom. The frequency values in red are promisingly *optimal* for each patient

All results of the 5 patients are shown in Fig. 4. Each of the square color frame presented the *CM* item at the specific frequency labeled below (the corresponding *GE* value was also listed following the frequency value). The x and y axes of the square frame were the channel number of the fNIRS, and the color at (*x*,*y*) indicated the value of the *CM*, which represented the connectivity strength between Channel-*x* and Channel-*y*. The higher the connectivity strength (the warmer the color), the lower the communication efficiency. Moreover, the global efficiency (*GE*) is defined to describe the overall communication efficiency. And the frequency corresponding to the coldest color distribution, i.e., the largest *GE* value, is promisingly optimal for each patient, respectively.

In addition to the global connectivity, local connection strengths of PFC, PL, and OL, i.e., *LS*_*PFC*_,*LS*_*PL*_, and *LS*_*OL*_, as well as the averaged local strength *LS*_*avg*_ were also calculated, as shown in Table 2. It can be seen that for each patient, the frequency at which the smallest average local connectivity was obtained was exactly the one where the highest GE was achieved. The results on local connectivity and global efficiency were consistent. Therefore, the frequencies of 125Hz, 130Hz, 100Hz, 160Hz, and 130Hz are promisingly optimal for patients P1, P2, P3, P4, and P5, respectively.

**Table 2:** The local functional connectivity analysis

Patient	Tested	Local strength				Global
	frequencies	*LS*_*PFC*_	*LS*_*PL*_	*LS*_*OL*_	*LS*_*aver*_	efficiency
P1	125Hz	0.3679	0.3524	0.1865	0.0306	2.8253
	100Hz	0.3217	0.4393	0.3969	0.1538	2.4961
	80Hz	0.3447	0.3591	0.4269	0.1339	2.3683
	60Hz	0.9445	0.9246	0.6804	1.0000	1.0446
P2	130Hz	0.1193	0.3374	0.1929	0.0693	3.4236
	160Hz	0.3052	0.3493	0.2356	0.4559	2.8045
	185Hz	0.3887	0.3382	0.1570	0.3672	2.7523
	100Hz	0.1966	0.4459	0.1874	0.6479	2.5749
P3	100Hz	0.0971	0.3370	0.2609	0.0467	4.0123
	80Hz	0.2135	0.3614	0.1784	0.1677	3.3139
	60Hz	0.4024	0.4137	0.1757	0.4549	2.3944
	125Hz	0.4348	0.6311	0.5208	1.0000	1.7739
P4	160Hz	0.3211	0.2568	0.3267	0.2162	3.3366
	130Hz	0.3493	0.3416	0.1224	0.3598	2.9649
	60Hz	0.4027	0.3632	0.0804	0.5598	2.7418
	80Hz	0.4260	0.4174	0.2646	0.9455	2.3951
P5	130Hz	0.1289	0.3812	0.3502	0.1330	3.5287
	80Hz	0.2989	0.3229	0.2711	0.2999	3.1407
	60Hz	0.3877	0.2994	0.5130	0.3953	2.7492
	160Hz	0.2315	0.4699	0.4734	0.7431	2.5838

### Comparison with clinical assessments

The proposed quantified assessment based on task fNIRS measurements and brain functional analysis was compared with clinical assessments including the on-site DBS programming decisions and post independent MDS-UPDRS ratings on the recorded videos of walking tests. The DBS programming decision, MDS-UPDRS ratings, and analysis on brain functional efficiency were conducted independently from each other, and the relative results were not revealed to the personnel performing other analysis until the entire study was accomplished. The results are shown in Table 3.

**Table 3:** Comparison of brain efficiency with clinical assessments including the on-site DBS programming decisions and post independent MDS-UPDRS ratings based on the recorded videos

Patient	DBS parameters			MDS-UPDRS scores ^*f*^			Brain global efficiency ^*g*^
	Electrode contact,		Optimal frequency				
DBS machines ^*a*^	voltage, and	Frequencies	determined by	S1	S2	Average	*GE*
	impulse duration ^*a*,*b*^		the doctors ^*c*,*d*,*e*^				
P1	Left hemisphere:						
	(10, 2.9V, 90 *μs*)	125Hz	125 Hz	1	1	1	2.8253
	(11, 2.7V, 60 *μs*)	100Hz		2	1	1.5	2.4961
Medtronic	Right hemisphere:	80Hz		2	2	2	2.3683
	(2, 2.7V, 60 *μs*)	60Hz		2	2	2	1.0446
	(3, 2.5V, 60 *μs*)						
P2	Left hemisphere:	130Hz	130 Hz	1	1	1	3.4236
	(6, 2.7V, 60 *μs*)	160Hz		1	1	1	2.8045
PINS	Right hemisphere:	185Hz		1	1	1	2.7523
	(2, 2.7V, 60 *μs*)	100Hz		1	1	1	2.5749
P3	Left hemisphere:						
	(10, 2V, 80 *μs*)	100Hz	100 Hz	1	1	1	4.0123
	(8, 2V, 60 *μs*)	80Hz		1	1	1	3.3139
Medtronic	Right hemisphere:	60Hz		1	1	1	2.3944
	(2, 1.7V, 60 *μs*)	125Hz		1	1	1	1.7739
	(3, 1.7V, 60 *μs*)						
P4	Left hemisphere:	160Hz	160 Hz	1	1	1	3.3366
	(9, 1.8V, 60 *μs*)	130Hz		1	1	1	2.9649
Medtronic	Right hemisphere:	60Hz		2	1	1.5	2.7418
	(3, 2.5V, 60 *μs*)	80Hz		2	1	1.5	2.3951
P5	Left hemisphere:	130Hz	130 Hz	1	1	1	3.5287
	(4, 3V, 60 *μs*)	80Hz		1	1	1	3.1407
PINS	Right hemisphere:	60Hz		1	1	1	2.7492
	(6, 3V, 60 *μs*)	160Hz		1	1	1	2.5838

^a^Definition of electrode contact points depends on the DBS machines

^b^The location and number of stimulation points were determined by the doctors

^c^These frequencies were the doctors’ on-site decision in DBS programming and actually used for treatment

^d^The optimal frequencies were determined by doctors considering the rigidity, tremor, gait and patients’ feeling. In the experiments, the optimal frequencies determined by doctors were consistent the frequencies that patients felt the most comfortable

^e^One-month follow-up after DBS programming reported that all the five patients were satisfied with the DBS treatment, and no adverse effect or feelings was reported or noticed by the patients, their families, or doctors

^f^The MDS-UPDRS scores were rated by two qualified and experienced doctors, based on the recorded videos on patient performance during DBS programming

^c,f,g^The DBS programming decision, MDS-UPDRS ratings, and GE analysis were conducted independently from each other, and the relative results were not revealed to the personnel performing other analysis until the entire study was accomplished

For all the five patients, the doctors’ on-site decision on DBS frequency were consistent with the optimal frequency by the brain functional connectivity analysis, i.e., the one that corresponds to both the highest global efficiency and lowest local connection strength. The doctors made the decisions according to the following clinical process [39, 40]: (1) assessing the rigidity and tremor of PD patients; (2) measuring the gait performance with MDS-UPDRS ratings; (3) communicating with patients about their comprehensive feeling such as comfortability, etc; and (4) choosing the best DBS frequency considering the rigidity, tremor, gait, and patients’ feeling. In the experiments, the doctors’ on-site decision on DBS frequency was consistent with frequencies that patients felt the most comfortable. One-month follow-up after DBS programming reported that all patients were satisfied with the DBS treatment, and no adverse effect or feelings was reported or noticed by the patients, their families, or doctors. The dopaminergic medication was unchanged during the one-month follow-up period.

MDS-UPDRS, the Unified Parkinson’s Disease Rating Scale by the Movement Disorder Society, has been universally used in clinical assessment on motor and non-motor aspects of Parkinson’s disease [41]. The videos of the five patients while performing the walking tests were sent to two qualified specialists, *S*1 and *S*2, who are experienced on gait assessment of PD patients and independent from this study. The MDS-UPDRS scores include 5 subratings, i.e., 0, 1, 2, 3, and 4, indicating *normal*, *slight*, *mild*, *moderate*, and *severe* symptoms, respectively. For all the five patients, both specialists gave the same MDS-UPDRS scores of 1, indicating slight movement disorder, for the patients’ walking performance under the applied DBS treatments that were also consistent with the best brain efficiency. This verified the efficacy of the DBS treatments and brain functional analysis. Nevertheless, the MDS-UPDRS scores were unable to further discriminate less significant differences in motor performance. For all the tested frequencies of patients P2, P4, and P5, both specialists gave the same MDS-UPDRS scores. For the performance differences that could be distinguished by the MDS-UPDRS scores, as patients P1 and P3, the results were all consistent with the brain functional analysis.

## Discussion

Starting from the fact that Parkinson’s disease is a neurological disorder and implicates motor performance, we proposed in this paper an objective and quantitative assessment method for DBS programming with task fNIRS measurements and brain functional connectivity analysis. To the best of our knowledge, this is the first fNIRS-based study on assessment and optimization of DBS therapy via recording of brain activation while performing motor tasks and analysis on global as well as regional brain efficiency. The methods were developed for post-operative DBS programming, but also has the potential for intra-operative assessment on correct targeting.

Brain functional analysis has been a long-time research focus of Parkinson’s disease. In [18], Rascol et al. measured the regional cerebral blood flow changes with PET during the execution of a finger-to-thumb opposition motor task in the cerebellar hemisphere of parkinsonian patients. Compared with healthy controls, Parkinson’s patients had increased brain activation in ipsilateral cerebellar hemisphere. In [42], Sabatini et al. analyzed the cortical change of PD patients in a complex sequential motor task with fMRI. Compared with normal controls, PD patients had a significant bilateral increase of fMRI signals in the primary sensorimotor cortex, lateral premotor cortex, inferior parietal cortex, caudal part of SMA, and anterior cingulate cortex. In [19], Zhang et al. analyzed the functional connectivity of ventral intermediate nucleus of thalamus (Vim) in tremor-dominant (TD) and akinetic-/rigid-dominant (ARD) PD patients with fMRI. In TD patients, the Vim nucleus had an increase of brain connectivity with dentate nucleus, primary motor cortex (M1), SMA, globus pallidus, premotor cortex, and parietal cortex compared with normal controls. In ARD patients, the Vim nucleus only exhibited increased connectivity with globus pallidus and limbic lobe compared with normal controls. In [20], Hou et al. evaluated the functional connectivity of default mode network (DMN) with resting-state fMRI data and found significantly increased connectivities of anterior DMN and prefrontal regions. The common finding of these studies is that PD patients are characterized with hypoactivation of SMA and hyperactivation of cortical motor regions (e.g., primary motor cortex, premotor cortex, parietal cortex) compared with normal controls. DBS can relatively normalize the hypoactivation of SMA and hyperactivation of other cortical regions and optimize the network profile toward healthy controls [43, 44]. Our findings are consistent with these studies. Effective DBS parameters could induce strong normalization and decrease the “extra” brain activation of PD patients in order to optimize the network profile toward healthy controls.

Gait performance is a primary concern for the patients and doctors. Therefore, we took the clinical 10-m walking test as the motor task for assessment, and accordingly, the DBS frequency as the varying parameter since it is directly associated with gait [28]. Other DBS parameters, the location of electrode contact, voltage amplitude, and impulse duration, as well as other motor and non-motor functions, are also important and can be addressed in future studies. The type and number of motor tasks are constrained by the patients’ physical condition.

Besides fNIRS, EEG can also measure brain activation for functional analysis of Parkinson’s disease, but typically for non-motor functions such as cognition [45] and emotion [46]. Technical challenges for EEG measurement during motor tasks include motion artifact and noise removal, source localization, fast set-up, etc. Although fMRI prohibits entry of DBS patients due to the electromagnetic fields, its excellent localization accuracy can facilitate brain functional analysis of PD patients without DBS for non-motor and motor tasks, as it has done to investigate rehabilitation induced brain reorganization after stroke with upper and lower extremity movements assisted by special mechatronic systems [47, 48].

A major limitation of this study is the small number of patients. Nevertheless, our experiment design and analysis method were based on the current understanding of the neurological mechanism of Parkinson’s disease, and the results were in line with this knowledge and also consistent across all the five patients. Moreover, our fNIRS system could calculate the results in 90.47 s, which is effective to assist the doctors in selecting the stimulus parameters. We hope this work can encourage more study, and more clinical evidence will promisingly enable quantified and individualized optimization of deep brain stimulation therapy for each Parkinson’s patient.

In the future, we will try to expand the number of patients and conduct a more detail investigation of DBS treatment optimization with different contacts, voltage, and pulse width. And we will try to explore more objective evaluation indicators and find appropriate gait analysis equipment for our experiments. Moreover, we will consider the change of total electrical energy delivered (TEED) [49, 50]. TEED is a comprehensive parameter considering frequency, voltage, pulse width, and impedance, which is directly related to the power consumption and battery drainage rate of implanted impulse generator.

## Conclusions

This was a pilot study on quantified assessment of DBS programming. For the first time, we recorded the brain signals of PD patients in clinical DBS programming process with a wearable fNIRS system and analyzed the collected signals for brain functional connectivity. Experimental results showed that fNIRS assessments and brain functional connectivity analysis promised an objective solution for patient-specific optimization of DBS treatment.

## Data Availability

Data that support the findings and software codes developed for the data analysis in this paper will be made available upon reasonable request to the corresponding authors.

## Abbreviations

DBS: Deep brain stimulation
PD: Parkinson’s disease
fNIRS: Functional near-infrared spectroscopy
PFC: Prefrontal cortex
PL: Parietal lobe
OL: Occipital lobe
*GE*: Global efficiency
*CM*: Connectivity matrix
